# Polarisation of Electron Density and Electronic Effects: Revisiting the Carbon–Halogen Bonds

**DOI:** 10.3390/molecules26206218

**Published:** 2021-10-14

**Authors:** Sébastien Menant, Frédéric Guégan, Vincent Tognetti, Lynda Merzoud, Laurent Joubert, Henry Chermette, Christophe Morell

**Affiliations:** 1 Institut des Sciences Analytiques, UMR 5280, CNRS, Université de Lyon, 5 rue de la Doua, F-69100 Villeurbanne, France; sebastien.menant@etu.univ-lyon1.fr (S.M.); lynda.merzoud@isa-lyon.fr (L.M.); henry.chermette@univ-lyon1.fr (H.C.); 2 IC2MP UMR 7285, Université de Poitiers-CNRS, 4 rue Michel Brunet TSA, CEDEX 9, 86073 Poitiers, France; 3 COBRA UMR 6014-FR 3038, Université de Rouen, INSA Rouen, CNRS, 1 rue Tesnière, CEDEX, 76821 Mont St Aignan, France; laurent.joubert@univ-rouen.fr

**Keywords:** electron polarisation, conceptual DFT, reactivity/selectivity descriptors, chemical bonding

## Abstract

Electronic effects (inductive and mesomeric) are of fundamental importance to understand the reactivity and selectivity of a molecule. In this article, polarisation temperature is used as a principal index to describe how electronic effects propagate in halogeno-alkanes and halogeno-alkenes. It is found that as chain length increases, polarisation temperature decreases. As expected, polarisation is much larger for alkenes than for alkanes. Finally, the polarisation mode of the carbon–fluorine bond is found to be quite different and might explain the unusual reactivity of fluoride compounds.

## 1. Introduction

Linus Pauling was one of the most prominent scientists in the 20th century. His contribution to theoretical chemistry is especially linked to his book “The nature of the chemical bond” published in 1933, cited several millions of times [1] in which, among many other contributions, he introduced a quantitative estimate of atom electronegativity. The scale he developed was based on thermodynamical data, and is still used for the semi-quantitative analysis of bonds. It must be underlined that in this usual model, electronegativity scales as a square root of an energy.

This concept has been instrumental since its inception to characterize chemical bonds. Indeed, bonds linking atoms of similar electronegativity will mainly be covalent, while they are expected to be more polar or more ionic when they involve elements with different electronegativity values. It is noteworthy that these concepts of covalence and ionicity of bonds can also be investigated from the valence bond point of view, a theory [2] that was strongly promoted by Pauling himself. Discussing the nature of bonds in a molecule remains a cornerstone in chemical interpretation, and while it can be basically tackled from Pauling’s electronegativity perspective, alternative approaches are possible.

Indeed, a few months later, Mulliken introduced another definition [3] for electronegativity, namely the average of ionization potential (IP) and electron affinity (EA). This scale has been less used because of the difficulty of experimentally measuring EAs at that time. By contrast with Pauling’s approach, within Mulliken’s scheme, electronegativity scales as an energy and is absolute. Over the years, other electronegativity scales have been also proposed, where it scales either as a force or a potential, or is dimensionless [4,5,6,7]. Some are even fairly recent [8,9].

Therefore, any relationship between all these scales can only be approximate, the fits being in general constrained to give values approaching 4 for fluorine, and 2 for hydrogen. A thorough description of these scales is reported in [10]. Indeed, Mulliken’s definition is just a numerical approximation (linearisation) of the opposite of the electronic chemical potential μ defined by Parr et al. in 1978 [11] within the framework of Density Functional Theory (DFT):

(1) $$\mu ={\left(\frac{\partial E}{\partial N}\right)}_{v(r)},$$

where *E* is the electronic energy, *N* the total number of electrons and v(r) the external potential.

This was the first chemical concept derived from DFT, giving rise to a bunch of indexes and concepts (hardness, linear response function (LRF)...) [12,13] making the so-called Conceptual DFT (C-DFT) a scientific area by itself. As previously mentioned, electronegativity allows characterize chemical bonds. We can thus expect various C-DFT descriptors to also be relevant to this purpose. More specifically, we will focus in the present paper on polarisation descriptors, which we recently derived using a time-independent Rayleigh–Schrödinger (RS)perturbation framework, and which we aim at applying to study electronic effects at stake in chemical bonds. Indeed, following his work on electronegativity, Pauling developed the notion of ubiquitous electronic effects (such as inductive and mesomeric ones) in chemical bonds, which are obviously connected to the idea of electron density polarisation as a response to an external perturbation (“what happens in place B when the electronic system is perturbed in place A”).

As a proof of concept, we have thus decided here to concentrate on halogen–carbon bonds, which may cover an interesting span of bonding types, since fluorine is the most electronegative element, whereas iodine features an electronegativity value close to that of carbon (2.66 and 2.55, respectively in Pauling’s scale). Accordingly, depending on halogen X, some C–X are predicted to range among the most polarised single bonds in organic chemistry, C–F showing the largest polarisation of all. Intuitively, one would then expect the C–F bond to be the most reactive in the series. Yet, experimentally C–F bonds are known to be much more inert than other halogen–carbon bonds, a feature that also reflects in the bond dissociation enthalpies (115, 84, 72, 58 kcal/mol for the H_3_CH_3_C bonds, X from F to I) [14]

From our point of view, C-DFT descriptors are hence tools of choice to unravel these different effects and to cast light on polarisation in these particular bonds. To this aim, this paper will be built as follows: in the next section, the basics for the description of polarisation within C-DFT will be briefly reviewed. The used theoretical methods will then bedescribed (Section 3), before an in-depth discussion of the results (Section 4).

## 2. Theoretical Background

Conceptual Density Functional Theory is a field of quantum chemistry in which one aims at understanding and rationalizing chemical rules through an electron density perspective [13,15,16]. In the last few years, a great deal of attention has been paid to the static linear response function (LRF) [17,18,19], which was shown to be effective to retrieve fundamental electronic effects such as inductive and mesomeric ones. The LRF is expressed as the first derivative of electron density ρ with respect to the external potential (as defined in Hohenberg–Kohn theory):

(2) $$\begin{array}{c} \chi \left(r,r^{\prime }\right)=\frac{\delta \rho (r)}{\delta v(r^{\prime })}=\frac{\delta \rho (r^{\prime })}{\delta v(r)}. \end{array}$$

This non-local kernel is to be interpreted as the variation of the electron density at point r when the external potential is changed at another location $r^{\prime }$ (and *vice versa* since this function is symmetric under the exchange of its own coordinates). Its connection with energy can be safely built using a second-order Taylor expansion of the electronic energy with respect to an infinitesimal change of the external potential at fixed electron number within the so-called *E*[*N*,*v*] canonical ensemble:

(3) $$\begin{array}{c} E=E_{0}+\int \rho \left(r\right)\delta v\left(r\right)dr+\frac{1}{2}\;\int \int \;\chi \left(r,r^{\prime }\right)\delta v\left(r\right)\delta v\left(r^{\prime }\right)drdr^{\prime }, \end{array}$$

where $E_{0}$ denotes the ground-state electronic energy of the unperturbed system. Very recently, this equation has been put forward through a statistical physics analysis of electronic polarisation [20]. Identifying the electronic cloud as the thermodynamic system of interest, the external potential can act as an external energy reservoir, susceptible to exchange both heat and work with the system. Then, it can be shown that the first-order correction to the energy (the first integral in the right-hand side in Equation (3)) can be seen as the work exchanged between the molecule and the perturbation:

(4) $$\begin{array}{c} \delta W=\delta E^{\left(1\right)}\left[\delta v(r)\right]=\int \rho \left(r\right)\delta v\left(r\right)dr. \end{array}$$

Still using the statistical physics perspective, the second-order correction to the energy corresponds to the heat exchange according to:

(5) $$\begin{array}{c} \delta E^{\left(2\right)}\left[\delta v(r)\right]=\frac{1}{2}\;\int \int \;\chi \left(r,r^{\prime }\right)\delta v\left(r\right)\delta v\left(r^{\prime }\right)drdr^{\prime }=\frac{1}{2}\int \delta \rho \left(r\right)\delta v\left(r\right)dr, \end{array}$$

which can be interpreted as a polarisation energy. Here, as we consider throughout this paper that no particle exchange occurs with the surroundings (in other words, the number of electrons remains conserved), the polarisation density integral over the space coordinates vanishes:

(6) $$\begin{array}{c} \int \delta \rho \left(r\right)dr=\;\int \int \;\chi \left(r,r^{\prime }\right)\delta v\left(r^{\prime }\right)drdr^{\prime }=0. \end{array}$$

To evaluate the reshuffling of the electron density, one can instead use the number of electrons that have been shifted by the polarisation induced by the external potential variation:

(7) $$\begin{array}{c} \delta N_{shifted}=\frac{1}{2}\int \left|\delta \rho \left(r\right)\right|dr. \end{array}$$

In practice, the best way so far to compute the static LRF is through the well-known Berkowitz–Parr formula that stems from traditional RS perturbation theory [21]:

(8) $$\begin{array}{c} \chi \left(r,r^{\prime }\right)=-\sum_{k=1}^{\infty }\frac{\rho_{0}^{k}\left(r\right)\rho_{0}^{k}\left(r^{\prime }\right)}{E_{k}-E_{0}}, \end{array}$$

where $\rho_{0}^{k}\left(r\right)$ is the transition density between the ground state and excited state *k* (we have here implicitly considered that all involved wavefunctions were real-valued, so that $\rho_{0}^{k}$ and $\rho_{k}^{0}$ are identical), i.e., the product of the ground-state wavefunction by the $k^{th}$ excited state wavefunction integrated over all spin coordinates and over all spatial coordinates but **r**. $E_{0}$ and $E_{k}$ are the energy of ground state and that of state *k*, respectively. It can be noticed that within this approximated form, the LRF is diagonal. This is not an “exotic” form since, as the LRF is a symmetric kernel, it can always be exactly diagonalised [22,23].

With this at hand, electron density polarisation and the associated energy [24] can be rewritten as

(9) $$\begin{array}{cc} \; & \delta \rho \left(r\right)=\int \sum_{k=1}^{\infty }\frac{\rho_{0}^{k}\left(r\right)\rho_{0}^{k}\left(r^{\prime }\right)}{E_{0}-E_{k}}\delta v\left(r^{\prime }\right)dr^{\prime }=\sum_{k=1}^{\infty }c_{k}\rho_{0}^{k}\left(r\right) \end{array}$$

(10) $$\begin{array}{cc} \; & \delta E^{\left(2\right)}\left[\delta v\left(r\right)\right]=\sum_{k=1}^{\infty }c_{k}^{2}\left(E_{0}-E_{k}\right) \end{array}$$

In summary, Equation (10) shows that everything goes as if the polarisation energy corresponds to the stabilisation energy the system experiences when the fraction of electron $c_{k}^{2}$ is promoted in the $k^{th}$ excited state of the unperturbed system, the (major) number of electrons remaining in the ground state being $c_{0}^{2}=1-\sum_{1}^{\infty }c_{k}^{2}$. The set of $c_{k}^{2}$ with $k\in \left(0,1,2,...,\infty \right)$ can be seen as the distribution of electrons in the perturbed system within the eigenstates of the unperturbed system. A polarisation spectrum can be defined by the representation of the distribution with respect to the excited states energies, $c_{k}^{2}=f\left(E_{k}\right)$ (see more details in our recent papers). A polarisation entropy can also be computed through the well-known Shannon formula:

(11) $$\begin{array}{c} \delta S_{pol}\left[\delta v(r)\right]=-k_{B}\sum_{k=0}^{\infty }c_{k}^{2}\times \ln\left(c_{k}^{2}\right). \end{array}$$

It should be noticed that while the LRF is an intrinsic property of the system, the polarisation density, polarisation entropy and polarisation energy are not, since they depend on the shape, orientation and position of the additional potential. However, these latter quantities can account for the evolution of an electron system when it is submitted to an external perturbation such as the approach of an electrophile or a nucleophile that can be simulated by such an additional potential. Moreover, as pointed out by Geerlings and De Proft, the LRF is somewhat cumbersome to deal with since it is function of two sets of spatial coordinates. Conversely, the electron density polarisation, the number of shifted electrons, the polarisation entropy and polarisation energy are either local or global quantities, hence much simpler to picture and more practical to use than a fully non-local kernel.

A temperature can also be defined as soon as one can calculate both a heat exchange and an entropy. The polarisation temperature reads:

(12) $$\begin{array}{c} T_{pol}=\frac{\delta E^{\left(2\right)}}{\delta S_{pol}}. \end{array}$$

As the derivative of two extensive quantities, the polarisation temperature is actually an intensive quantity. Therefore, it does not come as a surprise that wherever the external potential perturbation is located, there is a linear relationship between polarisation heat and polarisation entropy, the slope being the polarisation temperature. Contrarily to both polarisation energy and entropy, polarisation temperature allows a comparison between systems with a different number of electrons. It may be noted that such temperature is found to depend only on the magnitude of the perturbation, and not on its position in space—hence this quantity is a global descriptor of the system under study.

Lastly, we will discuss some special formulations of this perturbing external potential. The simplest ones considered here are a uniform static electric field (EF) and a point charge (that can be easily extended to a collection of point charges by a superposition principle). In the first case, the potential associated with a space-independent and time-independent infinitesimal EF, $\delta F_{c}$ = $\delta F_{c}\hat{u}$ (where $\hat{u}$ is a unit vector), is (up to an arbitrary additive constant) $\delta v_{F}\left(r\right)=-\delta F_{c}\cdot r$. Equation (3) then becomes:

(13) $$\begin{array}{c} E=E_{0}-\delta F_{c}\cdot \int \rho \left(r\right)rdr+\frac{\delta F_{c}^{2}}{2}\;\int \int \;\chi \left(r,r^{\prime }\right)\left(\hat{u}\cdot r\right)\left(\hat{u}\cdot r^{\prime }\right)drdr^{\prime }. \end{array}$$

The first integral in the right-hand side is no more than the electronic part of the molecular dipole moment $d_{e}$. For the sake of simplicity, we are now choosing $\hat{u}$ along the *z* axis, so that:

(14) $$\begin{array}{c} E=E_{0}-d_{e}\cdot \delta F_{c}+\frac{\delta F_{c}^{2}}{2}\;\int \int \;\chi \left(r,r^{\prime }\right)zz^{\prime }drdr^{\prime }. \end{array}$$

The traditional second-order Taylor expansion for the energy with respect to the EF is:

(15) $$\begin{array}{c} E\left(F\right)=E_{0}-d_{e}\cdot F+\alpha \frac{F^{2}}{2}, \end{array}$$

where α denotes the relevant component of the molecular polarisability tensor, which thus identifies to the second integral in Equation (14). The link between polarisability and LRF is then fully established and suggests that polarisability should also be considered to deal with bond polarity.

Finally, we consider an infinitesimal point charge perturbation δq at point $R_{c}$, generating an infinitesimal external potential $\delta v_{q}\left(r\right)=\delta q/\left|r-R_{c}\right|$. It is straightforward to see that the first-order correction, the work, is the product of δq by the electronic part of the molecular electrostatic potential. On the other hand, using the definition of the polarisation energy and the Berkowitz–Parr relationship (Equation (10)), one can evaluate the heat exchanged with the surroundings:

(16) $$\begin{array}{c} \delta E^{\left(2\right)}\left[\delta v_{q}\right]=\frac{\delta q^{2}}{2}\;\int \int \;-\sum_{k=0}^{\infty }\frac{\rho_{0}^{k}\left(r\right)\rho_{0}^{k}\left(r^{\prime }\right)}{\left(E_{k}-E_{0}\right)|r-R_{c}\left|\right|r^{\prime }-R_{c}|}drdr^{\prime }. \end{array}$$

It is plain to see that the integrated function is the product of one function depending only on r with another one depending on only $r^{\prime }$, so that the double integral can be simply written as the product of two simple integrals, which are, by symmetry, equal. The last equation thus simplifies:

(17) $$\begin{array}{c} \delta E^{\left(2\right)}\left[\delta v_{q}\right]=-\frac{\delta q^{2}}{2}\sum_{k=0}^{\infty }\frac{1}{E_{k}-E_{0}}{\left(\int \frac{\rho_{0}^{k}\left(r\right)}{|r-R_{c}|}dr\right)}^{2}. \end{array}$$

It immediately follows from this expression that the polarisation energy is then always negative, regardless of the sign of the point charge: this electron density polarisation definitely triggers a stabilisation of the electronic system.

## 3. Materials and Methods

All DFT calculations were performed using orca (rev. 3.0 and 4.0) [25]. Geometry optimisations were carried out without any constraints at the B3LYP/def2-SVP level, and frequency calculations conducted at the same level of theory to ensure no imaginary frequencies were present. The first 50 excited states were then computed under the Tamm–Dancoff approximation (TDA) [26], at the B3LYP/aug-cc-pVTZ level of theory. Large basis sets and diffuse functions are indeed often necessary to correctly model excited states. Conversely, their impact on geometries is often rather small, although they significantly increase computation time. Hence, optimisations with large basis sets become prohibitively long for the largest molecules in our study, suggesting a compromise needed to be found.

To assess the validity of our compromise (small basis set optimisation, large basis set for electronic properties), we ran additional calculations using the aug-cc-pVTZ basis set both for geometry optimisations and excited state computations for the smallest systems under study (carbon chains from 1 to 4 atoms). Satisfactorily, results matched those obtained using mixed basis sets.

Polarisation descriptors (energy, entropy, temperature) were then computed using a home-made Fortran90 program, which is available on request to the authors, using cube files for the transition densities (see Ref. [24] for the calculation details).

The LRF was computed in the so-called frozen molecular orbital approximation (actually corresponding to that of the fictitious non-interacting Kohn–Sham (KS) system) for closed-shell systems according to eq. 53 in the Geerlings–De Proft review: [27]

(18) $$\begin{array}{c} \chi \left(r,r^{\prime }\right)\approx -4\sum_{i,b}\frac{\phi_{i}\left(r\right)\phi_{b}\left(r\right)\phi_{i}\left(r^{\prime }\right)\phi_{b}\left(r^{\prime }\right)}{\epsilon_{b}-\epsilon_{i}}, \end{array}$$

where $\phi_{i}\left(r\right)$ denotes a doubly occupied KS molecular orbital (MO) with energy $\epsilon_{i}$ while $\phi_{b}\left(r\right)$ is a vacant (i.e., virtual) one. Atomic and diatomic condensation requires orbital overlaps (see eq. 86 in the previous reference), which were here computed within the framework of Bader’s Quantum Theory of Atoms-In-Molecules (QTAIM) [28] by our own implementation in the ADF software [29,30]. For these calculations, the ADF TZ2P Slater-type basis set was used.

Atomic polarisabilities in the molecules (also called “distributed polarisabilities”) were computed using the procedure developed by Macchi and co-workers. [31] In a nutshell, QTAIM atomic dipole moments were evaluated in the presence of a finite external uniform static EF (with the recommended magnitude equal to 0.050 atomic units) in the six possible directions (*x*, *−x*, *y*, *−y*, *z*, *−z*). The atomic polarisability tensor can then be reconstructed by finite linearisation. Mean values are finally estimated by taking one third of the trace of this tensor. Such calculations were performed using our own interface between the Gaussian09 [32] and AIMAll packages [33] at the B3LYP/aug-cc-pVTZ level of theory.

## 4. Results and Discussion

### 4.1. Studied Systems

**Figure 1:**
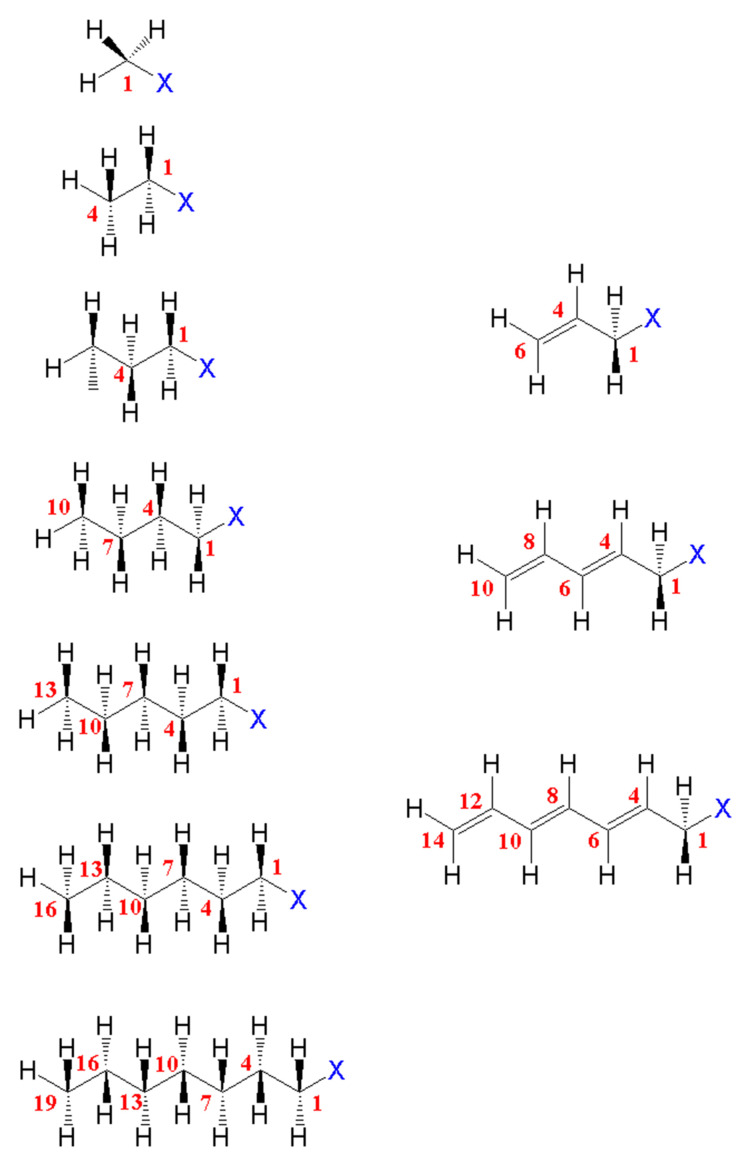
Studied molecules and numbering: halogeno-alkanes (**left**) and halogeno-alkenes (**right**).

The present investigation aims at exploring the inductive and mesomeric effects in halogeno-alkanes and halogeno-alkenes (see Figure 1). To achieve this goal, a set oflinear molecules have been computed. To allow unbiased comparison, the only considered halogens are fluorine, chlorine and bromine, since for iodine relativistic effects must be included.

To explore the electron donating/withdrawing effects of the halogen upon the carbon backbone, the external potential that polarises the electron density has been located either at the nucleus of the carbon bonded to the halogen or at the halogen itself. Polarisation densities, energies, entropies and shifted electrondensity have been then calculated with the potential settled this way, using 0.1*e* perturbing charge. After that, the potential has been successively shifted onto each nucleus of the molecule. It can be noticed that applyingthe perturbation directly at a nucleus allows oneto modifythe actual screening of the nucleus charge by the electron density and is also reminiscent of the H* method [34].

### 4.2. Saturated Compounds

**Figure 2:**
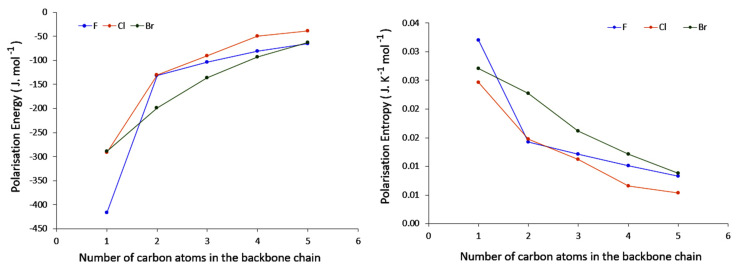
Evolution of the polarisation Energy (**left**) and Entropy (**right**) when the Carbon Neighbour to the Halogen is perturbed with respect to the number of carbon atoms in the backbone chain.

We present in Figure 2 the evolution of the polarisation energy and entropy for the various halogeno-alkanes.

As already stated, no comparison based upon both $\delta E^{\left(2\right)}$ and $\delta S_{pol}$ is possible since these two quantities are extensive. However, it is plain to see from Figure 2 that while chlorine and bromine derivatives happen to follow the same pattern, fluorine derivatives seem to follow a different one, especially for the shortest carbon chains. Another important tendency is that whatever the halogen derivative, it can be observed that both the polarisation energy and entropy are converging to similar values as the number of carbons in the backbone increases. The convergence appears to be achieved when the backbone is of 5 carbons.An interpretation of these two observations will be provided further on.

**Figure 3:**
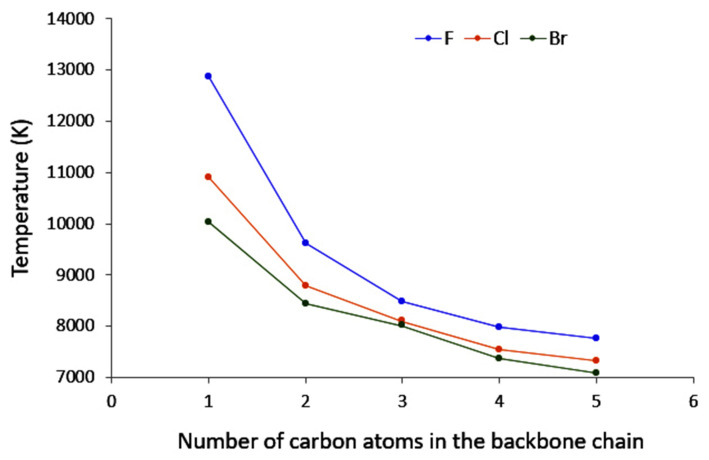
Evolution of the polarisation temperature with respect to the number of carbon atoms in the backbone chain when the Carbon Neighbour to the Halogen is perturbed.

The polarisation temperatures, represented in Figure 3 are indeed more straightforward to analyze. For each halogen, the polarisation temperatures tend to decrease as the carbon chain length increases. Noticeably, this decrease is diminishing along the series, suggesting that eventually temperatures may saturate to a constant value. This is in line with what has been observed for both polarisation energy and entropy. Some elements can be put forward to account for this. Indeed, polarisation temperature describes how easily a system may distort its electron density in response to given perturbation (the lower the temperature, the more polarisable the system is). In principle, perturbation by a negative point charge should result in an electron density displacement from the location of the point charge, and one may expect that the further away the displaced electron density can go, the lower the electrostatic repulsion. Hence, the larger the system, the lower the temperature. Now, of course the nature of the chemical system at hand must be taken into account. In the case of saturated compounds, electron density distortion will occur through the σ bond system, ultimately relying on inductive effects. It has been long anticipated that inductive effects strongly diminish along a carbon chain, hence it can be expected in first principles that polarisation effects should yield a plateau. This proposed explanation also rationalizes what has been observed for both polarisation energy and entropy.

**Figure 4:**
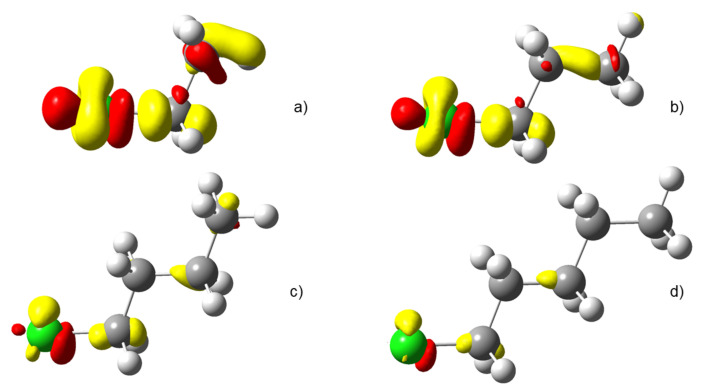
polarisation electron density of alkyl chlorides from (**a**–**d**), two to five carbons. δρ>0 colored in red while δρ<0 colored in yellow. Isovalue of $5.0\times 10^{-5}a.u.$

**Figure 5:**
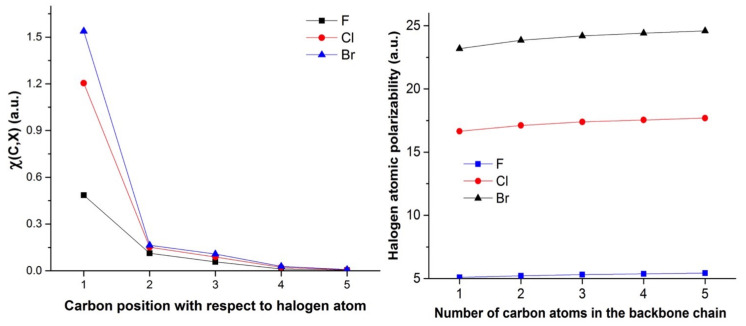
**Left**: Evolution for the QTAIM-condensed linear response kernel between halogen and the various carbon atoms. **Right**: Evolution of the atomic polarisability for halogen atom with respect to the alkyl chain length.

This simple interpretation is nicely corroborated by the shape of the electron density reshuffling isosurfaces depicted in Figure 4 (only alkyl chlorides are represented). In the case of the longest carbon chain (d), most of the electron density reorganisation is located within first three σ bonds from the perturbed nuclei. Actually, the density response barely reaches the fourth and is almost non-existent for the fifth. Figure 4 is quite a nice illustration of the well-known organic chemistry textbook rule: *butyl is futile*. Interestingly, a similar conclusion can be drawn by looking at the variation of the QTAIM-condensed linear response kernel between the halogen atom and each carbon atom, χ*(X,C)*, as represented in the left graph in Figure 5. As already noticed by Geerlings, De Proft and collaborators in related compounds, inductive effects decrease more quickly with respect to the distance between the two considered atoms, values for Cl and Br being slightly higher than those for F.

**Figure 6:**
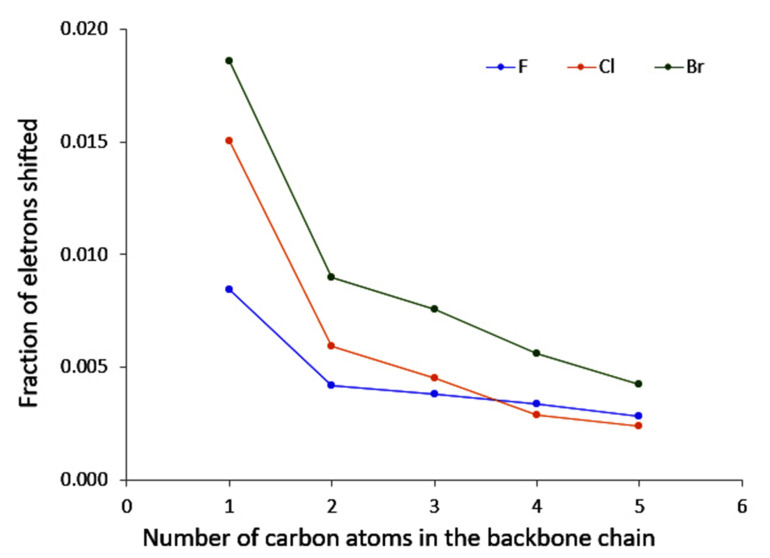
Evolution of the fraction of electron shifted when the carbon neighbour to the halogen is perturbed with respect to the number of carbon atoms in the backbone chain.

Now turning to the results for a given carbon chain length, an additional trend can be delineated. Indeed, we observe that temperatures for the fluoro derivatives are higher than those of the bromo and chloro compounds. In fact, it appears that the heavier the halogen, the lower the temperature. A comparable observation can be made for the shifted fraction of electron (see Figure 6 computed for a perturbation on the substituted carbon): chloro and bromo derivatives offer comparable behaviours, while the fluoro derivatives are rather systematically associated with a lower value.

Here too, some elements can be put forward to account for these observations. Indeed, we expect the polarisability of the halogens to increase with their atomic number. This is indeed the case as shown by the right graph in Figure 5. The distributed polarisabilities for the halogen atoms slightly increase with the carbon chain size and follows the F < Cl < Br expected trend. However, the three curves are fully separated and do not exhibit the crossings or convergence at some points observed for instance in Figure 2 and Figure 3. This is certainly due to the fact that these polarisabilities are computed assuming a uniform external electric field, which strongly differs from that generated by a point charge, which decreases with distance. One may even believe that uniform external EFs are actually a very poor model for the electric field created by a chemical environment that is in general anisotropic, so that such atomic polarisabilities should be, from our point of view, interpreted with caution. From our previous analysis, stronger responses to perturbation can be expected for the bromo and chloro derivatives, compared to fluoroalkanes—but at this stage nothing explains the clear differentiation of F from Cl and Br. A partial explanation is proposed in the following section.

### 4.3. C-Halides Polarisation Mode

**Figure 7:**
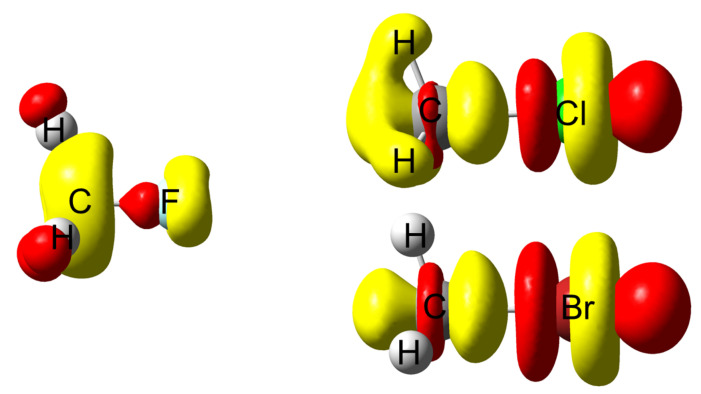
Electron density polarisation in methyl halides when the carbon neighbour to the halogen is perturbed; methyl fluoride (middle left), methyl chloride (upper right) methyl bromide (lower right). δρ>0 colored in red while δρ<0 colored in yellow. Isovalue of $2\times 10^{-5}a.u.$

In Figure 7 the density polarisation 3D maps of methyl halides are displayed. It is worth noticing that CH_3_Cl and CH_3_Br look quite alike while the density polarisation of CH_3_F exhibits a quite different feature. An investigation on the polarisation mode shows that only a few excited states display significant contributions to the perturbation response at the first carbon nucleus. For the heavier halides (Cl and Br), the most representative contribution is constructed by a loss of electron density at the first carbon atom and a gain at the halogen, along the interatomic axis (σ-orbital-type response). In the language of molecular orbital (MO) theory, polarisation in these cases is piloted by the promotion of a fraction of electron from a bonding σ(C–X) MO to the associated antibonding $\sigma^{⋆}\left(C–X\right)$. This assertion has been confirmed by a natural transition orbital (NTO) analysis (not reported here).

**Figure 8:**
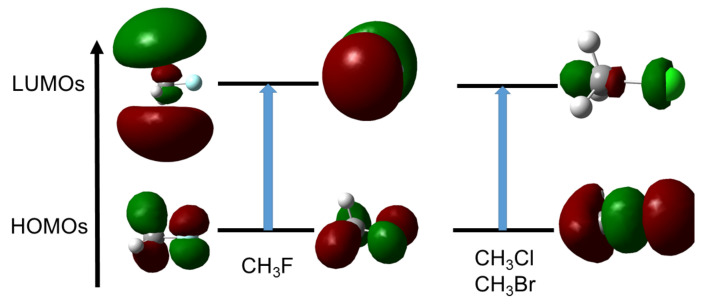
Orbitals involved in the density polarisation of CH_3_F (**left**) and CH_3_Cl (**right**). CH_3_Br follows the same pattern as CH_3_Cl.

This phenomenon provokes the weakening of the *C*–*X* bond and is certainly at the origin of the first stage of a first-order Nucleophilic Substitution ($SN_{1}$). The process that weakens the C–X bond by polarisation ends up with the carbon and halogen being drawn apart from one another due to the bond breaking. The polarisation pattern of methyl-fluoride turns out to be rather different. The response is developed perpendicular to the inter-nuclei axis as if the response were supported by a π-like system. Two different situations are encountered. In the case of CH_3_F, in MO terms the polarisation response is triggered by an electron promotion from an occupied “lone-pair” type π MO (constructed through a combination of a 2p(F) AO with a C–H contributions) to a similar MO relying on a 3p(C) AO. This is schematized in Figure 8 In the case of longer carbon chains, the “accepting” orbital is an antibonding $\sigma^{⋆}$(C–C) MO. In both cases, no significant weakening of the C–F bond is expected. This is perfectly in line with the lower reactivity ascribed to these bonds compared to other halogen–carbon bonds.

A more detailed study of the MO diagram of these alkyl halides helps to understand this strong difference in behaviour. Indeed, the bonding σ(C–X) MO is deeply buried in the case of CH_3_F, compared to CH_3_Cl and CH_3_Br. Promotion of an electron in this MO is thus severely hampered, and thus π-type response becomes preferred.

### 4.4. Unsaturated Compounds

**Figure 9:**
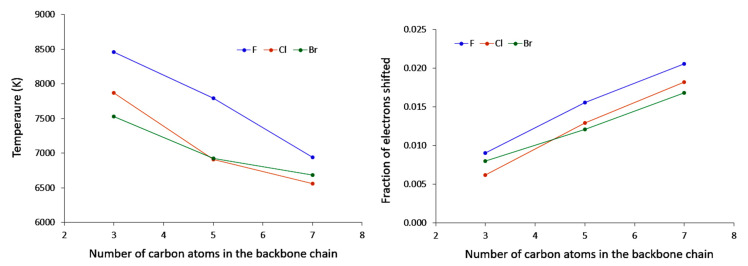
Evolution of the polarisation temperature and the fraction of electron shifted with respect to the number of carbon atoms in the backbone chain when the carbon neighbour to the halogen is perturbed.

If we now turn our attention to unsaturated compounds, some differences are demonstrated. As was observed for haloalkanes, temperatures are also decreasing for a fixed halogen and as the length of the carbon chain increases (see Figure 9). However, the same is not true for the shifted fraction of electrons; on the contrary, this descriptor is increasing (as proven by Figure 9).

Nevertheless, chloro and bromo derivatives once again present comparable features, while the fluoro compounds stand out.

**Figure 10:**
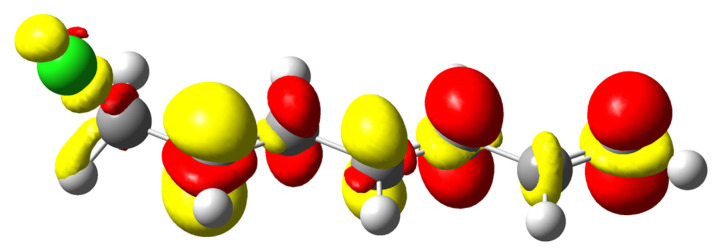
Polarisation electron density in 1-chloromethyl-hexatriene δρ>0 colored in red while δρ<0 colored in yellow. Isovalue of $1.0\times 10^{-4}a.u.$

Here also, some elements can be provided to account for these observations. Beside their inductive effects, halogens are also presenting mesomeric effects, which may be active in these compounds. Conjugation with the unsaturated chain thus allows electron density movements to spread over a rather large distance in the compounds, as can be seen in Figure 10. However, another feature is also evident from this Figure: besides the π-system response, an opposite response of the σ-backbone is present. From Equation (5), we may expect these “counter”-responses to mitigate the stabilisation from the π system reorganisation, and to relate to the inductive effects one may expect from halogens (inductive acceptor, mesomeric donor groups). This effect can be seen as an application at the electron level of the well-known *Le Chatelier’s rule* or as a molecular electronic *Lenz’s Law*. Hence, two opposite factors appear to be active in these cases:

- conjugation, which allows a larger electron density movement, reflecting in the larger $\delta N_{shifted}$ values compared to the saturated compounds (and the incident increase in their value with the elongation of the conjugated chain);
- “counter” polarisation of the σ backbone, stemming from the inductive effects of the halogen, and resulting in a slower decrease of temperature than could be expected.

To a lesser extent, these effects seem to be also present for halogeno-alkyls, but as expected induction in such cases prevails over mesomerism.

## 5. Conclusions

In a fairly recent paper [20], several new descriptors such as the polarisation temperature have been derived from a statistical physics view of density polarisation. In the present contribution, these indexes have been used to investigate how electronic effects develop and propagate in halogeno-alkanes and halogeno-alkenes. As expected, the investigation has shown that the longer the carbon backbone, the more polarisable the molecule. Moreover, it has been found that the density polarisation is barely measurable beyond four carbons for an alkyl chain while it develops further for alkenes. This confirms the organic chemistry rule *butyl is futile*. A sort of *Le Chatelier rule* for halogeno-alkenes has also been observed. Indeed, as the density polarisation propagates through the π bonding system, the σ bond backbone reacts to counterbalance the density reshuffling. Finally, for both halogeno-alkanes and halogeno-alkenes, the polarisation mode is different for fluorine than that of the other halogens. This difference in pattern might be at the origin of the unusual chemistry of fluoride derivatives.

## Data Availability

The Fortran code for the calculation of all the indexes is available on demand from the corresponding authors.

## Sample Availability

Samples of the compounds are available from the authors.

## Abbreviations

The following abbreviations are used in this manuscript:

AO	atomic orbital
C-DFT	Conceptual Density Functional Theory
DFT	Density Functional Theory
EA	electron affinity
EF	electric field
IP	ionization potential
KS	Kohn–Sham
LRF	Linear Response Function
MO	molecular orbital
NTO	Natural Transition Orbital
QTAIM	Quantum Theory of Atoms-in-Molecules
RS	Rayleigh–Schrödinger
TDA	Tamm–Dancoff Approximation
